# Editorial: Exploring the power of AI and ML in smart grids: advancements, applications, and challenges

**DOI:** 10.3389/frai.2025.1615547

**Published:** 2025-06-09

**Authors:** Vikram Kulkarni, Sarat Kumar Sahoo, Bhushankumar Nemade, Suresh Kallam, Chakkrit Termritthikun

**Affiliations:** ^1^Department of Information Technology, Mukesh Patel School of Technology Management and Engineering, SVKM's NMIMS (Deemed to be University), Mumbai, Maharashtra, India; ^2^Department of Electrical Engineering, Parala Maharaja Engineering College (P.M.E.C), Biju Patnaik University of Technology (BPUT), Berhampur, Odisha, India; ^3^Department of Computer Science Engineering, Shree L.R. Tiwari College of Engineering, Mumbai University, Mumbai, Maharashtra, India; ^4^Department of Computer Science Engineering (IoT), JAIN (Deemed-to-be University), Bengaluru, Karnataka, India; ^5^School of Renewable Energy and Smart Grid Technology (SGtech), Naresuan University, Phitsanulok, Thailand

**Keywords:** artificial intelligence, machine learning (ML), smart grid (SG) technologies, renewable and sustainable energy, cyber security

**Figure 1:**
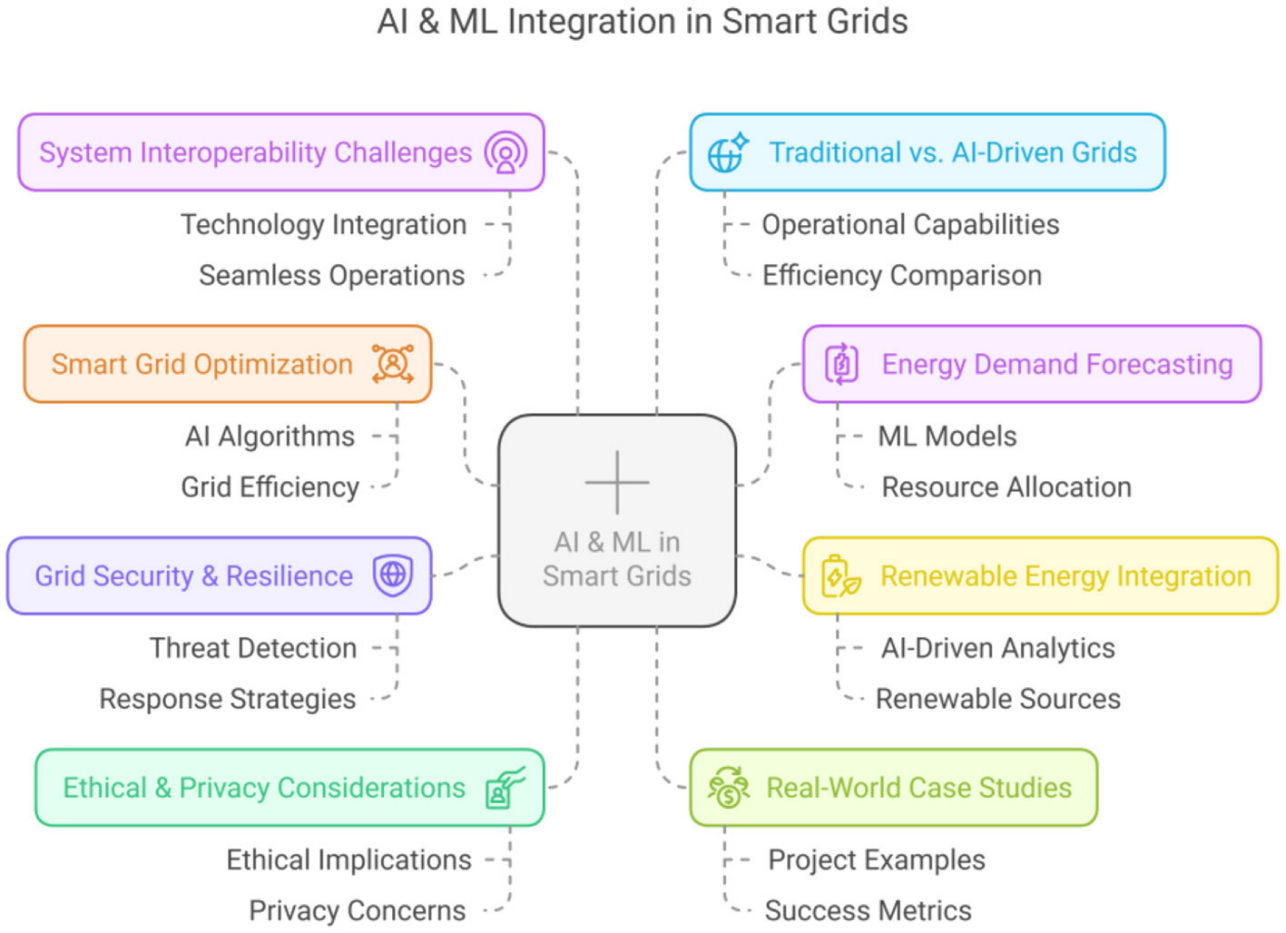
AI & ML integration in smart grids: key areas and outcomes.

Artificial Intelligence (AI) and Machine Learning (ML) technologies are significantly transforming the operation and management of energy grids worldwide. Smart grids, featuring advanced communication systems, intelligent sensor networks, renewable energy integration, and sophisticated control mechanisms, stand at the forefront of this technological evolution. This Research Topic compiles cutting-edge research highlighting critical advancements, diverse applications, and significant challenges related to leveraging AI and ML technologies to enhance smart grid infrastructures. The key areas and outcomes are presented in Figure 1.

This Research Topic is entitled “*Exploring the power of AI and ML in smart grids: advancements, applications, and challenges*.” It aims to assemble innovative research and pragmatic solutions employing AI and ML techniques within energy systems, demonstrating the current and future roles of these technologies in enhancing energy system operations. Contributions sought were intended to vividly highlight diverse applications, advancements, and associated challenges of integrating AI and ML into smart grids. This Research Topic serves as a crucial resource to comprehend the present status and future directions of AI and ML in smart grid technologies. To ensure that AI-based smart grids are interoperable, secure, and capable of protecting data privacy, standard frameworks such as IEC 61850 define communication protocols for intelligent electronic devices in electrical substations. Furthermore, the General Data Protection Regulation (GDPR) promotes responsible data management, establishing the foundation for scalable and secure smart grid deployment.

Topics specifically covered within this Research Topic include but are not limited to:

- Innovations in AI and ML for smart grid optimization.
- AI-driven energy demand forecasting and management.
- ML applications in renewable energy integration.
- AI solutions for smart grid security and resilience.
- Case studies on AI/ML applications enhancing grid stability and efficiency.
- Comparative studies between traditional and AI/ML-based smart grid systems.
- Ethical and privacy concerns in AI/ML applications in smart grids.
- Challenges related to integrating AI/ML into smart grid systems, including data security and system interoperability.

Energy forecasting is pivotal in smart grid technology, directly impacting the efficiency and reliability of energy management. Badhe et al. introduced an advanced predictive model integrating the Temporal Fusion Transformer (TFT) with the Aquila Optimizer (AO). Their model demonstrated enhanced accuracy and computational efficiency, significantly outperforming traditional forecasting models. This research underscores the necessity for precise forecasting mechanisms to effectively manage renewable energy variability and dynamic energy consumption patterns.

Shrivastava and Goswami presented a hybrid neuro-fuzzy deep learning model tailored specifically for optimizing building energy management systems (BEMS). Implemented within a university setting, their IoT-driven model showcased substantial practical efficiency by achieving a remarkable 20% reduction in electricity bills. The model effectively utilized 2 years of collected data to forecast and optimize energy use, emphasizing AI's critical role in cost-effective energy management in resource-constrained economies.

Cybersecurity remains a vital aspect of smart grid technology. Nemade et al. proposed a comprehensive cyber-defense strategy employing deep learning techniques to significantly enhance cybersecurity, particularly targeting vulnerabilities in SCADA systems within smart grids. Their approach demonstrated robust anomaly detection, significantly strengthening infrastructure resilience. By integrating advanced graph-based algorithms and human-AI interaction strategies, their work highlights the urgency and importance of advanced cybersecurity measures to safeguard increasingly digitalized energy infrastructures.

Further emphasizing cybersecurity, Verma and Rao explored the application of deep learning in decentralized smart grid architectures. Their research provided essential insights into identifying and mitigating threats inherent to distributed energy resources. This study illustrated the efficacy of AI-driven cybersecurity solutions, critical for protecting decentralized infrastructures against sophisticated and emerging cyber threats.

Addressing the unique implementation challenges in developing regions, Talhar Belge et al. conducted a comprehensive review highlighting advancements, challenges, and future opportunities for smart grid technology in India. Their research underscored the importance of digital communication systems, advanced metering infrastructure, and real-time grid management for enhancing reliability and efficiency. Through practical case studies and pilot project analyses, the authors outlined a strategic roadmap for smoother transitions from traditional grids to smarter, more resilient energy systems.

Mahadik et al. focused on environmental sustainability enhanced through smart sensors. Their review illustrated how sensor technologies significantly improve real-time monitoring, fault detection, and renewable energy integration, leading to reduced greenhouse gas emissions and optimized resource management. Their findings underscore the pivotal role of intelligent sensor technologies in achieving broader sustainability goals within smart grids.

Balamurugan et al. provided a concise yet thorough overview of AI applications in smart grids, detailing methods from load forecasting to power distribution optimization and renewable energy integration. Their review underscored AI's vast potential for enhancing grid reliability, operational efficiency, and adaptability to future energy demands. Additionally, they addressed critical challenges such as data quality, standardization, and interoperability, highlighting the strategic significance of AI for future grid operations.

Collectively, the research contributions within this Research Topic underscore substantial opportunities and persistent challenges involved in integrating AI and ML into smart grid systems. Ongoing advancements in AI technology, robust cybersecurity frameworks, and adaptive energy management systems remain crucial for exploiting the transformative potential of these technologies. While significant progress has been made, continuous innovation is essential to address evolving complexities and maximize the benefits of AI-driven smart grid solutions.

Looking forward, substantial research opportunities remain to further leverage AI and ML in smart grid technologies. Future research could explore hybrid optimization techniques combining multiple AI algorithms to enhance prediction accuracy and computational efficiency. Real-time adaptive AI models, capable of dynamically responding to varying grid conditions, represent another promising research direction. Additionally, developing scalable, cost-effective AI solutions tailored for resource-constrained environments prevalent in developing countries is critical. Continuous advancements in cybersecurity frameworks through proactive and evolving threat detection mechanisms are equally essential.

This Research Topic serves as an essential resource for researchers, industry professionals, and policymakers, highlighting current innovations and identifying areas for further exploration. We anticipate that this editorial fosters ongoing innovation and collaboration within the dynamic field of AI-driven smart energy management, ultimately supporting the transition toward more sustainable and efficient global energy ecosystems.

